# Lung cancer awareness training experiences of community health workers in KwaZulu-Natal, South Africa

**DOI:** 10.4102/phcfm.v14i1.3414

**Published:** 2022-12-09

**Authors:** Siyabonga B. Dlamini, Khumbulani W. Hlongwana, Themba G. Ginindza

**Affiliations:** 1 Discipline of Public Health Medicine, Faculty of Health Sciences, University of KwaZulu-Natal, Durban, South Africa; 2 Cancer & Infectious Diseases Epidemiology Research Unit, College of Health Science, University of KwaZulu-Natal, Durban, South Africa

**Keywords:** lung cancer, community awareness, community health workers, prevention, training experiences

## Abstract

**Background:**

Lung cancer is the leading cause of cancer mortality worldwide. Awareness interventions in the developing world remain scarce. Community health workers (CHWs) are a critical component towards ensuring efficient delivery of healthcare services in low- and middle-income countries.

**Aim:**

This study explored the experiences of CHWs of their training as lung cancer awareness intervention implementers.

**Setting:**

The study was conducted in a resource-poor setting, with CHWs from previously disadvantaged communities.

**Methods:**

On the last day of training, 10 CHWs were requested to voluntarily participate in a focus group discussion regarding their experiences of the training, utilising a discussion guide.

**Results:**

The participants expressed positive experiences with the training. They cited the amenable and conducive learning environment established by the facilitator. The participants felt empowered through the newly acquired knowledge and wanted to help their communities. However, some participants expressed a desire to have other forms of learning incorporated in future training. The participants were also cognisant of existing gaps in their own knowledge that could be elaborated upon in preparation for potential questions by the community. Some participants confirmed their role as agents of change.

**Conclusion:**

The authors propose large-scale intervention studies of lung cancer awareness utilising the CHW programme to gather conclusive evidence regarding their effectiveness at a community level.

**Contribution:**

This article provides insight into the training of community health workers on lung cancer awareness and future research on the integration of the intervention into already existing programmes.

## Introduction

Community health workers (CHWs) remain a critical component towards ensuring efficient delivery of healthcare services in low- and middle-income countries (LMICs). They are integral in the prevention, treatment and care of individuals living with various disease conditions, such as human immunodeficiency virus (HIV) and acquired immunodeficiency syndrome (AIDS), childhood illnesses and tuberculosis (TB), as well as provision of palliative care services through home-based care.^1,2,3^ The provision of these services by CHWs has been especially vital in remote areas, where inaccessibility to health facilities is a challenge.

In South Africa, the services offered by CHWs are included within a primary health care (PHC) ward-based outreach team. This team consists of a professional nurse, environmental health practitioner, health promotion practitioner and four to five CHWs.^4^ These teams have since been experiencing varying degrees of success throughout the country.^5,6,7,8,9,10,11,12,13,14^ It could be argued that potentially there could be a risk of overwhelming the CHWs with health interventions at community level. However, the CHWs have been instrumental in combating TB infection in South Africa and other countries of similar contexts through the directly observed treatment and therapy programme,^3,15,16,17,18,19^ apart from their other responsibilities. Evidently, TB shares similarities in terms of signs and symptoms with lung cancer, hence poor treatment response by their patients misclassified as TB patients.^20,21^

The authors’ project, as part of a larger study of lung cancer in sub-Saharan Africa, introduced the training of CHWs on lung cancer community awareness intervention, as a contribution to promoting early lung cancer screening, detection and care. In South Africa, lung cancer is among the top four ranking cancers in terms of morbidity and mortality after breast, prostate and cervical cancers.^22^ Among men, lung cancer is the leading cause of cancer deaths in the country, while it ranked third among women.^23^ However, the trends of lung cancer mortality in Africa are based on scanty epidemiological evidence,^24^ and the burden of disease may be underestimated.^25,26^ Poor health outcomes have been documented for lung cancer patients, including death while undergoing treatment.^27^ More than 80% of lung cancer cases are linked to cigarette smoking and other tobacco products,^28^ therefore making a case for expanded prevention efforts at the community level. Community health workers are better placed to carry out this function at this level. To the authors’ knowledge, there are limited published studies on lung cancer health interventions by CHWs at the community level. In addition, it has been established that currently, the CHW curriculum does not include lung cancer awareness.^29,30^ Hence, the opportunity to work with CHWs in raising awareness on lung cancer at community level was considered an important contribution to potentially ensuring early disease detection, diagnosis and treatment, as CHWs often serve as linkages between health facilities and patients. Therefore, the integration of lung cancer awareness interventions on the training of CHWs should be explored. This article explored the CHW experiences of the lung cancer awareness intervention training in a South African province.

## Research methods and design

### Study design

This was a qualitative study conducted among CHWs who attended the training on lung cancer. This study used phenomenology to explore the training experiences of CHWs about lung cancer.^31^ The aim of phenomenology is to understand the meanings that people make of the world in their everyday lives. This is normally done through discovering meanings in people’s conversations or text. In this case, the conversation was in the form of a focus group discussion (FGD).

### Study setting

Community health workers were recruited from the selected communities in the two metropolitan cities in a South African province. These communities consisted of townships where previously disadvantaged populations dwell and a suburb. Initial meetings to introduce the project were held with the local ward councillors. The ward councillors offered their cooperation and assisted with identifying the CHWs who could potentially work in the project as data collectors (a baseline paper on lung cancer knowledge, attitudes and practices is currently under review) and project implementers. They were in possession of lists of CHWs who were awaiting placement on community projects. Given the political nature of ward councillors, the received names of CHWs were subjected to stringent selection criteria and the successful CHWs attended training on lung cancer awareness.

### Logistics

The period of the training was weeklong. The CHWs travelled to a central location and resided at a training venue for the duration of the training. All subsistence expenses were supported through the project funds. Prior to the commencement of training, the CHWs completed a questionnaire to assess their level of knowledge of lung cancer and pitch the training at an appropriate level but also to incorporate additional concepts into the training materials. The questionnaire contained sociodemographic details, questions on their knowledge of lung cancer signs and symptoms, their attitudes towards lung cancer and their health-seeking behaviour when suspecting lung cancer. Upon completion of the training, they completed the same questionnaire to observe any change in their level of knowledge. The results of these assessments will be presented elsewhere. Tablets were used to administer the questionnaire. The questionnaire took approximately 20 min to complete. The REDCap software (Vanderbilt University, Nashville, Tennessee, United States) was most appropriate because of the accessibility of the platform even in areas with no Internet access and password protection of the data.

### Training of community health workers on the intervention

The researcher conducted the training during the 5 days using a printed training manual which was provided to each CHW on the first day. This manual was adapted from the Lung Laboratory – Research and Intervention Centre’s lung cancer training manual, developed by pulmonologists in the field. The following content was included: (1) background and lung cancer in the South African context; (2) types of lung cancer; (3) how lung cancer affects the body; (4) stages of lung cancer; (5) signs and symptoms of lung cancer; (6) causes and risk factors of lung cancer; (7) diagnosis, management and treatment of lung cancer; and (8) referral pathways of lung cancer patients. The training also addressed vital steps for consideration by CHWs during the implementation of the intervention in their respective communities.

Smoking, as the major risk factor for lung cancer documented by the literature,^32,33^ was discussed during the training, including the benefits and processes of quitting smoking. The information, education and communication (IEC) materials included for the training were in English. During training, these contents were translated into the predominant local language by the facilitator, who is also the researcher, with the assistance of CHWs to ensure clarity and understanding. These translations were included in the information package provided to the CHWs for implementation purposes. Role plays on the delivery of the content at the household level were included on the fourth and fifth days of training, which afforded reinforcing learning content.

### Study population and sampling strategy

During the selection process of the CHWs, approximately 50 names were provided by ward councillors as potential participants. They had to have attained at least a Grade 12 qualification to be selected in the project, and a total of 25 CHWs were selected to participate in the training.

The researcher met with all the identified CHWs to introduce the project. Of the 25 selected CHWs, the majority (*n* = 21) were female. Two young men eventually retracted from the project. They were introduced to the processes of training on a series of topics (including data collection for baseline survey on lung cancer awareness, lung cancer community awareness intervention and data collection for the postintervention survey) during the project. This article focuses on their experiences during the community lung cancer awareness intervention training. These experiences were explored through a FGD.

### Data collection

On the last day of training, 10 CHWs were identified to voluntarily participate in a FGD regarding their experiences of the training. These participants had a wide range of work experience, and the group was mixed in age, including both male and female participants to allow for sufficient depth and multiple perspectives to emerge. They were considered information-rich to adequately address the topic of interest. This FGD took place in the same room used for training in the absence of other CHWs for privacy. Although the FGD was facilitated by a novice CHW, who also participated in the training to ensure inclusivity and openness during the discussion, she was trained on FGD and other necessary research processes pertinent to qualitative research. Furthermore, the lead researcher had worked with this group of CHWs in prior training, which provided an opportunity of building and consolidating rapport with the group. The CHW worked under close supervision of the experienced research team. The lead author had prior training and experience in qualitative methodologies, with one co-author involved in the teaching and supervision of master’s- and PhD-level students using qualitative research methods. The CHW FGD facilitator was apprised of the study aim and the discussion guide as a data collection tool. A debriefing session was conducted after she completed the facilitation and the FGD had ended. She utilised the discussion guide to engage with the group. The group discussion was conducted in both English and a local language for one hour. The researcher was not present during the discussion. The group discussion with permission from the participants was recorded, transcribed and thereafter translated into English. The transcription and translation were done by an experienced research assistant. The researcher checked the transcript and translation against the original recording for quality.

### Data analysis

The English transcript was analysed by the researcher using NVivo version 12 (QSR International, Burlington, Massachusetts, United States). The researcher used thematic analysis for emergent themes. Thematic analysis is a method often used for identifying, analysing and understanding patterns of meaning emerging from qualitative data.^34,35^ Although this method has been deemed devoid of theoretical commitments, its application has been demonstrated across a variety of theoretical frameworks and research paradigms.^35^ In this study, thematic analysis was used to identify patterns in relation to the CHWs’ lived experiences regarding the lung cancer awareness training they participated in, following an inductive approach.^36^ Chunks of data were coded as themes that emerged from the data.

### Ethical considerations

This study obtained ethics approval from the University of KwaZulu-Natal Biomedical Research Ethics Committee (BREC) (reference number BF585/18) and gatekeeper permission from the local ward councillors. The informed consent process was explained, emphasising the voluntariness of their participation and that if they chose not to participate, they would not face any penalties. Thereafter, they signed the informed consent form. Confidentiality was ensured through anonymising the participants’ responses in the transcript and assigning numbers to them.

## Results

A final total of 23 CHWs participated in the training, after two potential CHWs retracted from the 25 initial selected. Ten participants (six women and four men) volunteered to participate in the FGD. Their ages ranged from 20 to 52 years. The majority were women who resided in the communities identified by the project (Table 1). The majority of the CHWs did not have postmatric qualifications. A few possessed postmatric training (e.g. a certificate-level training).

**TABLE 1:** Demographic profile of the community health workers participating in the focus group discussion regarding their experiences of lung cancer training.

Variable	Number	Percentage
**Gender**		
Female	6	60
Male	4	40
**Age (in years)**		
< 25	2	20
25–30	2	20
31–35	3	30
> 35	3	30
**Marital status**		
Single	6	60
Married or living with a partner	2	20
Widowed, divorced or separated	2	20
**Educational status**		
Matric (Grade 12)	10	100
Postmatric (certificate, diploma, degree)	5	50
**Race or ethnicity**		
Black people	8	80
Mixed-race people	2	20

**FIGURE 1:**
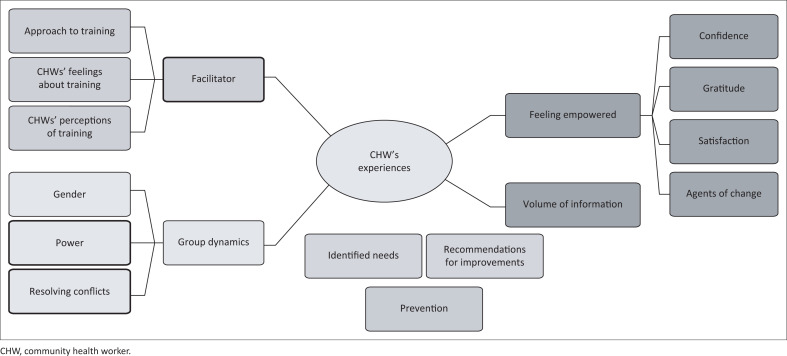
Thematic representation of community health workers’ experiences of lung cancer awareness training in KwaZulu-Natal.

Figure 1 provides a descriptive summary of the emergent themes that emanated from the FGD data of the CHWs’ experiences of the lung cancer awareness training.

### Facilitator-related factors

The approach of the researcher, who was also the training facilitator, to the training was deemed engaging and acceptable in promoting understanding of all participants, particularly in being cognisant of the various levels of CHWs’ knowledge. There was consensus on his accessibility and effort to ensure understanding of contents:

‘The facilitator was very understanding in explaining to everybody and making sure that everyone understood what he was trying to portray, and also his style was very easy to understand … He even made some drawings on the board to explain more what he was doing and what he was trying to say.’ (Participant 6, male, 23 years)

‘The way the teacher taught us was good, and when you asked a question, he didn’t have a correct and wrong answer. It was equal, so even when you came with an idea, he would listen. Even when you came with a question that was not based on the training, he would answer, so everything was good.’ (Participant 5, female, 32 years)

The CHWs were trained by professionals who were also proficient in the local languages, which facilitated understanding of the various concepts included in the training. Despite the CHWs’ overall appreciation of the style of facilitation, some offered alternatives for inclusion to enhance the modality of teaching and learning:

‘I think what would help is that as much as we are learning the book, it would help also if whilst learning we could be doing some practicals at the same time so that when we teach other people, we teach them something we know and understand. So that by the time we go to the community, we already know it and understand it better.’ (Participant 4, female, 34 years)

These experiences emphasise the various CHW needs for consideration during training. Individuals effectively learn through various approaches and media, as supported by our results. While the majority communicated the extensive knowledge acquired, others suggested more attention to reinforce learning as essential. Participants communicated their positive experience by citing the amenable and conducive learning environment established by the facilitator:

‘I felt that he was extremely approachable; you could ask him anything you want, basically just feel at home and feel very welcome.’ (Participant 2, male, 23 years)

‘The way he taught us, he made us all able to ask questions without fear, when you were not clear … ’ (Participant 1, female, 43 years)

Such a learning environment established the CHWs as valued partners in the learning experience. They suggested no inferiority was evident and they were viewed as active participants in their own learning process.

### Group dynamics

Participants alluded to the gender dynamic since the majority of the CHWs were female and the training facilitator was a man in his early 40s. One participant valued the difference and appreciated the professionalism in coping with discord or disagreement within the group.

‘[…*W*]hat I saw, considering he is a male teaching a class full of women where sometimes there are conflicts as women, but he was able to handle all that because he was the head …’ (Participant 4, female, 34 years)

Culture seems to have played a role on how the participants viewed and interpreted their relationship with the training facilitator. Culturally, a man is accepted as the head of the household and therefore is expected to lead. This expectation was not communicated explicitly. However, this role of headship was naturally afforded to the training facilitator, which was positively experienced.

The inherent power dynamics were captured by a CHW whose observation of the training facilitator’s leadership skills was valued. It is evident that the dichotomy of the teacher–learner was appreciated rather than an egalitarian relationship within the context of engagement. The amenable approach to conflict resolution was acknowledged:

‘[…*E*]veryone reported to him; if there was something he heard, he wouldn’t come harshly to talk to you and tell you not to do something, and he was able to handle situations well.’ (Participant 4, female, 34 years)

‘[…*T*]he way he treated his learners and the way he conducted himself as a teacher. So the friendship he built trying to bond with his learners. I thought it was very good, which makes one see their way forward.’ (Participant 9, male, 30 years)

### Acquisition of empowering knowledge

The value of expanding their own knowledge to further engage purposefully with the community was supported by the data:

‘[…*T*]he community didn’t know much about the lung cancer; they know there are cancers, but they are not able to differentiate them, and the lung cancer is the one they don’t understand much. They just ask you questions even you don’t know how you will answer. But now I think with everything I have learnt, I will be able to give answers.’ (Participant 4, female, 34 years)

‘The training was very informative and really taught us a lot about lung cancer; it taught us a lot of things we didn’t know and a lot of things that now we can teach other people…’ (Participant 6, male, 23 years)

The confidence of their acquired knowledge incrementally capacitated them towards advocacy:

‘My biggest wish is that we could go to the companies, especially the ones that are using these things that cause cancer, to do awareness and double-check whether they really do get all the uniform, how their safety measures are for the people working there. Even in construction companies, we sometimes see people working without safety gear…’ (Participant 9, male, 30 years)

Their self-assurance through their expanded knowledge was expressed and justified:

‘I feel very confident right now. I feel like I could be a doctor [*they laugh*], because that’s how much information we learnt about lung cancer. Everything was so good…’ (Participant 6, male, 23 years)

In addition to the self-assurance, they conveyed their gratitude of being an inextricable part of the solution in their communities:

‘I am grateful, and I am 100% that I can do what I have to do for my community.’ (Participant 4, female, 34 years)

### Community health workers as agents of change

Some CHWs confirmed their role as agents of change:

‘[…*T*]he importance of knowing that cancer can be cured. It’s not like you have a death disease, you have a death sentence; you can get back up and survive, there are a lot of people who have survived … If we could bring back hope that they can still live and be all right.’ (Participant 10, female, 38 years)

‘I am very proud of what I am about to do, and I’m very proud that I will go out to the community to help people with this information that I got.’ (Participant 3, female, 34 years)

### Volume of information

The volume of information covered during the training and its comprehensiveness was observed and could potentially be overwhelming:

‘I think there was definitely not a shortage of information, maybe at times a bit too much …’ (Participant 2, male, 23 years)

Others were satisfied with the volume of content:

‘I think the information we got was good and even though we still need to know more about lung cancer … we are satisfied with what we got for now, the information we have available.’ (Participant 7, female, 36 years)

### Community health workers’ identified needs

The participants were also cognisant of existing gaps in their own knowledge that could be elaborated upon in preparation for potential questions by the community:

‘I think what we have learnt is all good, but we wish for the next session we will have to incorporate other cancers because in the community we get a lot of questions about a lot of things we don’t know.’ (Participant 5, female, 32 years)

One participant reflected on a specific and pervasive aspect of cravings for cigarettes and how they can help:

‘I wish to know more so that we can be able to help our brothers who are in trouble with cigarettes, who are in trouble with cravings, what can we do to end the cravings, so we need to know more of what I can do for them. Maybe with them too it’s not easy when we come and say cigarette this and that, we also need to have a plan that we can come up with, “OK, maybe if you can use this when maybe the cravings start.”’ (Participant 8, male, 28 years)

### Recommendations for improvements based on the training

There were definitive areas of improvement identified by the CHWs. Based on the training, the CHWs felt strongly that the targeted areas could be expanded to maximise the effect of the awareness campaign:

‘I wish we could go out to rural areas, because now what tends to happen is that only Durban gets taught, but in other areas they know nothing. Like in prisons, you see, in prisons there are sick people, but there are no people advising them on how to handle this. They smoke cannabis, cigarettes and all that.’ (Participant 8, male, 28 years)

‘[…*W*]ill echo Number 1, who mentioned that we need to go to the schools, to the kids in school. I think that even in primary schools, we can go whilst the kids are still young so that when they grow up, they would already have knowledge about what actually happens with cancer. What actually happens with a person who smokes. In high schools we can go. The children there are looking at their brothers who smoke and they also want to do that, but if as they grow, they know that cigarettes are dangerous …’ (Participant 8, male, 28 years)

‘[…*M*]aybe to places like the clubs, although we might not be able to go in, because in there, there is a lot of smoking happening and different kinds of substances – because what we are fighting is lung cancer. Warning them about cigarettes, it would be great to continue more with our outreaches and reach the small kids so that in the end we don’t end up with a nation that has a huge lung cancer problem …’ (Participant 3, female, 34 years)

Another participant recommended specific aspects of the training sessions on including assessments:

‘I suppose what I would have done differently is maybe the time frame, the length of the training and also, perhaps, added in some more written tests on certain subjects regarding lung cancer.’ (Participant 2, male, 23 years)

Clearly, the above participant preferred continued engagement on the contents to gauge his knowledge acquisition.

## Discussion

This study showed that CHWs had, on the whole, positive experiences of training, and this seemed to have encouraged CHWs to be a positive change in their communities. The training facilitator’s approach to the training was embraced positively. This fostered positive interactions and a positive learning environment. Consequently, this environment could increase the likelihood of the CHWs performing their tasks more competently in their communities. A study conducted in Bangladesh, Mexico, Guatemala and South Africa assessing the CHWs’ ability to screen for cardiovascular disease revealed the CHWs were able to conduct their tasks at levels comparable to the formally trained health professionals.^37^

Although most of the CHWs highly appreciated the knowledge gained during training, some of the CHWs suggested using alternate modes of learning as reinforcement. A study mapping the evidence from various CHW trainings demonstrated an increasing trend on the use of mobile technologies to support training in LMIC contexts.^38^ However, this trend was not widely adopted for broad training, service delivery interventions and supervision at the community level involving CHWs. In settings where Internet connectivity and mobile data availability are not a challenge, these mobile technologies could be a viable platform for providing training and support to the CHWs. However, South Africa may not be able to provide such platforms and service, especially in the rural areas where CHWs services are most valuable.^39,40,41^ For these technologies to be implemented in South Africa, the Internet infrastructure needs to be made widely accessible and be stable for use in the rural areas of the country.

The CHWs were encouraged to be active participants in their own learning, thus enforcing a positive learning environment. A study conducted on the training of CHWs regarding oral health intervention indicated that the CHWs performed well on their knowledge scores after training.^42^ When questioned about the presenters leading the training, the CHWs also communicated a positive opinion. Similarly, they felt that the level of the training together with its duration were ideal. These results seem to align with the findings of the present study. However, a study conducted among CHWs in Cape Town on noncommunicable diseases and training concluded that the knowledge on the subject matter was poor while their training was unstandardised and haphazard.^43^

Although culture seems to have played a role in the group dynamics, it is not clear whether a male training facilitator would be preferred in the future trainings among this group of individuals. Future studies could explore this phenomenon and inform the successful training of CHWs. Studies have investigated how culturally adapted interventions implemented by CHWs affect service provision.^44,45,46,47,48^

The dynamics of power between the group and the researcher were perceived positively, which seem to have contributed to the learning environment. These findings affirm that if there is a relationship of trust and collegiality, it fosters a good learning and working environment. VeneKlasen and Miller^49^ offer that power relationships do not have to be one-dimensional; they are viewed as toxic and unchanging. These relationships can also be collaborative and transformative (i.e. power with). The efforts of sharing of power were noted by participants. A study conducted in South Africa regarding interpersonal trust among CHWs and their supervisors indicated the importance of this concept.^50,51^ Because the CHWs’ supervisors were perceived negatively in terms of their relationship with the CHWs, this seemed to affect the CHWs’ performance at work. By extension, this performance could even spill over to their training and learning environment. Nonetheless, the acknowledgement of the brokenness of these by those in power usually leads to the willingness to work together in resolving them and moving forward.^50^

The feeling of empowerment through the knowledge gained from the training was seen as a confidence booster to engage with the community regarding lung cancer. A study conducted in rural Cape Town on self-management training among CHWs indicated that the CHWs expressed confidence about what they learnt and motivated them to change or modify certain behaviours detrimental to their own health.^52^ Although CHWs participating in the present study hardly viewed the newly acquired knowledge in relation to their own health, they still considered that information would help them better perform their tasks as CHWs. Some suggested that their motivation to engage in training was their desire to assist others. Similar sentiments were also conveyed in a study that evaluated CHW training outcomes in two districts of KwaZulu-Natal, which revealed that the CHWs had marked improvements on their knowledge scores.^53^ They also reported high levels of contentment with their training and discernible increases in their confidence in advising clients. However, increases in the level of knowledge after training does not always translate to confidence providing services that they are trained for. A Brazilian study on the training of CHWs regarding cervical cancer screening alluded to this phenomenon.^54^ It has also been demonstrated that poor training and supervision often results in poor knowledge retention and job performance.^43^ Invariably, this could also affect feelings of being empowered and motivated to perform on the job.

The CHWs’ roles of being advocates and agents of change were not necessarily motivated by politics or progressivism, as has been reported in other studies,^55,56^ although it could be suggested that visiting companies where employees are deemed to be at risk of lung cancer, doing uniform inspections and assessing whether the safety protocols are observed is a form of activism. In this case, it seems to be the desire to assist their community members and those deemed at risk of lung cancer in avoiding the dire consequences of not attending to the disease early, suggesting an altruistic motivation.^55,57,58^ Being the agent of change in this case means effecting behaviour change or being part of the process of achieving desired outcomes at the community level.^59^

Some of the CHWs had strong feelings about going to the places of entertainment to raise awareness about risk factors of lung cancer. The South African government has put restrictions and prohibitions in the advertisement of tobacco products in public spaces, including clubs.^60^ Therefore, these actions would be responding to the government’s prerogative of reinforcing the regulations. It is not clear, however, how this could be accomplished in the clubs, as the club owners have a right to restrict what happens in their places of business. This kind of awareness could possibly be done outside the club as the patrons are waiting to go inside the club.

L.M. English and colleagues investigated the trends of cigarette use among the youth in South Africa and Botswana using the Global Youth Tobacco Survey (GYTS) data.^61^ They demonstrated a significant decreasing trend of tobacco use over time in South Africa. However, this trend was no longer significant when factoring the parental use of cigarettes. In Botswana, the trend was the opposite of the South Africans’, where there was an observed upward trend over time. This highlights the importance of intervening early, as mentioned by the participant above.

Studies conducted in LMICs regarding assessments of training outcomes of CHWs have varying success of knowledge retention among CHWs.^62^ Half of the studies used pre- and post-training assessments and the other half added a follow-up test 6–8 months after training. In our study, pre- and post-training assessments were done. These assessments were used as a measure to gauge the CHWs’ knowledge increase because of the training, not in the traditional sense where students write a test and are graded. However, the results are not part of the scope of this article. The current study gives important insights on the approach and content of a lung cancer awareness training programme for CHWs and the integration of such training to existing training curricula. Nonetheless, the results are from the experiences of a limited number of CHWs. Finally, researchers’ experiences in qualitative research, establishment of rapport between the lead researcher and the research participants, training of the FGD facilitator, use of debriefing sessions between the FGD facilitator and lead researcher, use of verbatim quotes in the presentation of the results, as well as clear audit trail of the research process all contributed to the trustworthiness of the study findings.

## Conclusion

Overall, the CHWs’ training experiences seemed to be positive. The group session afforded acknowledgment of the gains in knowledge and closing of the existing gaps among CHWs. They offered definitive thoughts on improvements and identified strategies that could be utilised in the process. The training seemed to have succeeded in empowering CHWs to perceive themselves as agents of change in the communities. The advocacy role was pivotal in sustaining learning. Although gender and age differences were acknowledged between the training facilitator and the participants, it positively influenced the interactional nature of learning. This component warrants further investigation.

Some studies have reported on the importance of follow-up training to reinforce learning.^43,63,64,65^ Future trainings of this group of health workers should consider other modes of training and reinforcing learning, such as the use of technology.

The CHWs recognise the significance of their training in facilitating their engagement and knowledge awareness of lung cancer in the communities. Evidently, they affirmed that early detection through awareness was a vital component of training in reducing the number of lung cancer cases. Their expressed commitment to such awareness was notable. The various sections of their 1-week training proved extensive and adequate in preparing them for further engagement with their communities of service. Hence, it would be prudent to consider integrating this modality of training and learning in the CHW TB training curriculum, because of the overlap in symptomatology between the two diseases and the misdiagnosis of lung cancer for TB. However, large-scale intervention studies are required to arrive at conclusive evidence on the effectiveness of such interventions and wide-scale adoption of such.

This article provides insight into the training of community health workers on lung cancer awareness and future research on the integration of the intervention into already existing programmes

## References

[1] Perry HB, Zulliger R, Rogers MM. Community health workers in low-, middle-, and high-income countries: An overview of their history, recent evolution, and current effectiveness. Annu Rev Public Health. 2014;35(1):399–421. 10.1146/annurev-publhealth-032013-182354 24387091

[2] Perry H, Zulliger R. How effective are community health workers. Baltimore: Johns Hopkins Bloomberg School of Public Health; 2012.

[3] Prasad BM, Chadha SS, Thekkur P, et al. Is there a difference in treatment outcome of tuberculosis patients: Rural healthcare providers versus community health workers? J Fam Med Prim Care. 2020;9(1):259–263. 10.4103/jfmpc.jfmpc_729_19 PMC7014860 32110601

[4] National Department of Health. Provincial guidelines for the implementation of the three streams of PHC re-engineering. Pretoria: South African National Department of Health; 2011.

[5] Doherty T, Tabana H, Jackson D, et al. Effect of home based HIV counselling and testing intervention in rural South Africa: Cluster randomised trial. Br Med J. 2013;346:f3481. 10.1136/bmj.f3481 23766483 PMC3685510

[6] Grimwood A, Fatti G, Mothibi E, Malahlela M, Shea J, Eley B. Community adherence support improves programme retention in children on antiretroviral treatment: A multicentre cohort study in South Africa. J Int AIDS Soc. 2012;15(2):17381. 10.7448/IAS.15.2.17381 22713255 PMC3499784

[7] Horwood C, Butler L, Barker P, et al. A continuous quality improvement intervention to improve the effectiveness of community health workers providing care to mothers and children: A cluster randomised controlled trial in South Africa. Hum Resour Health. 2017;15(1):39. 10.1186/s12960-017-0210-7 28610590 PMC5470211

[8] Le Roux IM, Tomlinson M, Harwood JM, et al. Outcomes of home visits for pregnant mothers and their infants: A cluster randomized controlled trial. AIDS. 2013;27(9):1461–1471. 10.1097/QAD.0b013e3283601b53 23435303 PMC3904359

[9] Loeliger KB, Niccolai LM, Mtungwa LN, Moll A, Shenoi SV. ‘I have to push him with a wheelbarrow to the clinic’: Community health workers’ roles, needs, and strategies to improve HIV care in Rural South Africa. AIDS Patient Care STDS. 2016;30(8):385–394. 10.1089/apc.2016.0096 27509239 PMC4991585

[10] Loeliger KB, Niccolai LM, Mtungwa LN, Moll A, Shenoi SV. Antiretroviral therapy initiation and adherence in rural South Africa: Community health workers’ perspectives on barriers and facilitators. AIDS Care. 2016;28(8):982–993. 10.1080/09540121.2016.1164292 27043077 PMC4917424

[11] Ndou T, Van Zyl G, Hlahane S, Goudge J. A rapid assessment of a community health worker pilot programme to improve the management of hypertension and diabetes in Emfuleni sub-district of Gauteng Province, South Africa. Glob Health Action. 2013;6:213–218. 10.3402/gha.v6i0.19228 PMC3556684 23364086

[12] Rotheram-Borus MJ, Tomlinson M, Le Roux IM, et al. A cluster randomised controlled effectiveness trial evaluating perinatal home visiting among South African mothers/infants. PLoS One. 2014;9(10):e105934. 10.1371/journal.pone.0105934 25340337 PMC4207699

[13] Tomlinson M, Doherty T, Ijumba P, et al. Goodstart: A cluster randomised effectiveness trial of an integrated, community-based package for maternal and newborn care, with prevention of mother-to-child transmission of HIV in a South African township. Trop Med Int Health. 2014;19(3):256–266. 10.1111/tmi.12257 24433230

[14] Thomas LS, Buch E, Pillay Y. An analysis of the services provided by community health workers within an urban district in South Africa: A key contribution towards universal access to care. Hum Resour Health. 2021;19(1):22. 10.1186/s12960-021-00565-4 33602255 PMC7889710

[15] Chowdhury AMR, Chowdhury S, Islam MN, Islam A, Vaughan JP. Control of tuberculosis by community health workers in Bangladesh. Lancet. 1997;350(9072):169–172. 10.1016/S0140-6736(96)11311-8 9250184

[16] De Grave D, Heunis CJ1, Kigoz GN, Peresu E. Patient satisfaction with directly observed treatment and multidrug-resistant tuberculosis injection administration by lay health workers in rural Eswatini. Afr J Prim Health Care Fam Med. 2020;12(1):1–10. 10.4102/phcfm.v12i1.2257 PMC7284164 32501027

[17] Howell EM, Kigozi NG, Heunis JC. Community-based directly observed treatment for TB patients to improve HIV services: A cross-sectional study in a South African province. BMC Health Serv Res. 2018;18(1):255. 10.1186/s12913-018-3074-1 29625569 PMC5889613

[18] Peresu E, Heunis CJ, Kigoz GN, De Grave D. Patient satisfaction with directly observed treatment and multidrug-resistant tuberculosis injection administration by lay health workers in rural Eswatini. Afr J Prim Health Care Fam Med. 2020;12(1):e1–e10. 10.4102/phcfm.v12i1.2257 PMC7284164 32501027

[19] Wright J, Walley J, Philip A, et al. Direct observation of treatment for tuberculosis: A randomized controlled trial of community health workers versus family members. Trop Med Int Health. 2004;9(5):559–565. 10.1111/j.1365-3156.2004.01230.x 15117299

[20] Keikha M, Esfahani BN. The relationship between tuberculosis and lung cancer. Adv Biomed Res. 2018;7:58. 10.4103/abr.abr_182_17 29657943 PMC5887688

[21] Bhatt M, Kant S, Bhaskar R. Pulmonary tuberculosis as differential diagnosis of lung cancer. S Asian J Cancer. 2012;1(1):36–42. 10.4103/2278-330X.96507 PMC3876596 24455507

[22] Fitzmaurice C, Allen C, Barber RM, et al. Global, regional, and national cancer incidence, mortality, years of life lost, years lived with disability, and disability-adjusted life-years for 32 cancer groups, 1990 to 2015: A systematic analysis for the global burden of disease study. JAMA Oncology. 2017;3(4):524–548. 10.1001/jamaoncol.2016.5688 27918777 PMC6103527

[23] Sung H, Ferlay J, Siegel RL, et al. Global cancer statistics 2020: GLOBOCAN estimates of incidence and mortality worldwide for 36 cancers in 185 countries. Cancer J Clin. 2021;71(3):209–249. 10.3322/caac.21660 33538338

[24] Islami F, Torre LA, Jemal A. Global trends of lung cancer mortality and smoking prevalence. Transl Lung Cancer Res. 2015;4(4):327–338. 26380174 10.3978/j.issn.2218-6751.2015.08.04 PMC4549470

[25] Ferlay J, Soerjomataram I, Dikshit R, et al. Cancer incidence and mortality worldwide: Sources, methods and major patterns in GLOBOCAN 2012. Int J Cancer. 2015;136(5):E359–E386. 10.1002/ijc.29210 25220842

[26] Van Eeden R, Tunmer M, Geldenhuys A, Nayler S, Rapoport BL. Lung Cancer in South Africa. J Thoracic Oncol. 2020;15(1):22–28. 10.1016/j.jtho.2019.06.032 31864550

[27] Denton E, Conron M. Improving outcomes in lung cancer: The value of the multidisciplinary health care team. J Multidiscip Healthc. 2016;9:137–144. 10.2147/JMDH.S76762 27099511 PMC4820200

[28] Alberg AJ, Brock MV, Ford JG, Samet JM, Spivack SD. Epidemiology of lung cancer: Diagnosis and management of lung cancer, 3rd ed: American College of Chest Physicians evidence-based clinical practice guidelines. Chest. 2013;143(Suppl 5):e1S–e29S. 10.1378/chest.12-2345 23649439 PMC4694610

[29] Janse van Rensburg MNS, Marcus TS. Evaluating community health worker education policy through a National Certificate (vocational) primary health qualification lens. Afr J Prim Health Care Fam Med. 2020;12(1). 10.4102/phcfm.v12i1.2104 PMC7061226 32129653

[30] KwaZulu-Natal Department of Health. Community health workers [homepage on the Internet]. KwaZulu-Natal Department of Health; 2001 [cited 19 July 2021]. Available from: http://www.kznhealth.gov.za/chw.htm

[31] Ritchie J, Lewis J, Nicholls CM, Ormston R. Qualitative research practice: A guide for social science students and researchers. Los Angeles, CA: Sage; 2013.

[32] Institute for Health Metrics and Evaluation. Global burben of disease 2015 [homepage on the Internet]. Washington, DC: Institute for Health Metrics and Evaluation; 2015 [cited 17 November 2021]. Available from: http://vizhub.healthdata.org/gbd-compare/#

[33] O’Keeffe LM, Taylor G, Huxley RR, Mitchell P, Woodward M, Peters SAE. Smoking as a risk factor for lung cancer in women and men: A systematic review and meta-analysis. BMJ Open. 2018;8(10):e021611. 10.1136/bmjopen-2018-021611 PMC6194454 30287668

[34] Braun V, Clarke V. Thematic analysis. APA handbook of research methods in psychology, Vol 2: Research designs: Quantitative, qualitative, neuropsychological, and biological. APA handbooks in psychology®. Washington, DC: American Psychological Association, 2012; p. 57–71.

[35] Clarke V, Braun V. Thematic analysis. J Posit Psychol. 2017;12(3):297–298. 10.1080/17439760.2016.1262613

[36] Holmqvist K, Frisén A. ‘I bet they aren’t that perfect in reality’: Appearance ideals viewed from the perspective of adolescents with a positive body image. Body Image. 2012;9(3):388–395. 10.1016/j.bodyim.2012.03.007 22542634

[37] Gaziano TA, Abrahams-Gessel S, Denman CA, et al. An assessment of community health workers’ ability to screen for cardiovascular disease risk with a simple, non-invasive risk assessment instrument in Bangladesh, Guatemala, Mexico, and South Africa: An observational study. Lancet Glob Health. 2015;3(9):E556–E563. 10.1016/S2214-109X(15)00143-6 26187361 PMC4795807

[38] Winters N, Langer L, Nduku P, et al. Using mobile technologies to support the training of community health workers in low-income and middle-income countries: Mapping the evidence. BMJ Glob Health. 2019;4(4):e001421. 10.1136/bmjgh-2019-001421 PMC6673767 31413872

[39] Lembani R, Gunter A, Breines M, Dalu MTB. The same course, different access: The digital divide between urban and rural distance education students in South Africa. J Geogr High Educ. 2020;44(1):70–84. 10.1080/03098265.2019.1694876

[40] Sithole MM, Moses C, Davids YD, et al. Extent of access to information and communications technology by the rural population of South Africa. Afr J Sci Technol Innov Dev. 2013;5(1):71–84. 10.1080/20421338.2013.782144

[41] Statistics South Africa. General household survey 2018. Pretoria: Statistics South Africa; 2018. Contract No.: P0318.

[42] Lumsden C, Andrews H, Leu C-S, Edelstein B. Changes in knowledge and beliefs of community health workers following an oral health intervention training program. J Prev Interv Community. 2019;47(1):54–65. 10.1080/10852352.2018.1547309 30806193

[43] Tsolekile LP, Schneider H, Puoane T. The roles, training and knowledge of community health workers about diabetes and hypertension in Khayelitsha, Cape Town. Curationis. 2018;41(1):1–8. 10.4102/curationis.v41i1.1815 PMC6091590 29781697

[44] Abrahams-Gessel S, Denman CA, Montano CM, et al. The training and fieldwork experiences of community health workers conducting population-based, noninvasive screening for CVD in LMIC. Glob Heart. 2015;10(1):45–54. 10.1016/j.gheart.2014.12.009 25754566 PMC4356015

[45] Eluka NN, Morrison SD, Sienkiewicz HS. ‘The wheel of my work’: Community health worker perspectives and experiences with facilitating refugee access to primary care services. Health Equity. 2021;5(1):253–260. 10.1089/heq.2020.0150 33937612 PMC8082038

[46] Falgas-Bague I, Ramos Z, Del Cueto P, et al. Adaptation of an evidence-based intervention for disability prevention, implemented by community health workers serving ethnic minority elders. Am J Geriatr Psychiatry. 2021;29(3):260–269. 10.1016/j.jagp.2020.07.014 32855041 PMC7855421

[47] Gittings L. ‘He can’t say a man’s stuff to a woman…’: Perspectives on the peferences of men living with HIV for gender-concordant care workers in Cape Town, South Africa. Men Masc. 2019;22(5):751–777. 10.1177/1097184X17732607

[48] Kowitt SD, Emmerling D, Fisher EB, Tanasugarn C. Community health workers as agents of health promotion: Analyzing Thailand’s village health volunteer program. J Commun Health. 2015;40(4):780–788. 10.1007/s10900-015-9999-y 25744815

[49] VeneKlasen L, Miller V. Power and empowerment. PLA Note. 2002;43:39–41. 10.3362/9781780444208.004

[50] Assegaai T, Schneider H, Scott V. Developing a district level supportive supervision framework for community health workers through co-production in South Africa. BMC Health Serv Res. 2021;21(1):337. 10.1186/s12913-021-06350-2 33853606 PMC8045385

[51] Assegaai T, Schneider H. Factors associated with workplace and interpersonal trust in the supervisory system of a community health worker programme in a Rural South African District. Int J Health Policy Manag. 2021;11:31–38. 10.34172/ijhpm.2021.03 PMC9278395 33619931

[52] Majee W, Anakwe A, Johnson L, Rhoda A, Frantz J, Schopp L. A self-management training intervention: Perceptions and practices of community health workers in South Africa. Health Promot Pract. 2019;21(6):983–992. 10.1177/1524839918820038 30616400

[53] Plowright A, Taylor C, Davies D, Sartori J, Hundt GL, Lilford RJ. Formative evaluation of a training intervention for community health workers in South Africa: A before and after study. PLoS One. 2018;13(9):e0202817. 10.1371/journal.pone.0202817 30248100 PMC6152868

[54] Kienen N, Bittencourt L, Pelloso SM, et al. Cervical cancer screening among underscreened and unscreened Brazilian women: Training community health workers to be agents of change. Res Educ Action. 2018;12(1):111–119. 10.1353/cpr.2018.0026 29755054

[55] Colvin CJ, Swartz A. Extension agents or agents of change? Community health workers and the politics of care work in postapartheid South Africa. Ann Anthropl Pract. 2015;39(1):29–41. 10.1111/napa.12062

[56] Schaaf M, Warthin C, Freedman L, Topp SM. The community health worker as service extender, cultural broker and social change agent: A critical interpretive synthesis of roles, intent and accountability. BMJ Global Health. 2020;5(6):e002296. 10.1136/bmjgh-2020-002296 PMC7299037 32546585

[57] Dageid W, Akintola O, Saeberg T. Sustaining motivation among community health workers in AIDS care in Kwazulu-Natal, South Africa: Challenges and prospects. J Community Psychol. 2016;44(5):569–585. 10.1002/jcop.21787

[58] Akintola O. What motivates people to volunteer? The case of volunteer AIDS caregivers in faith-based organizations in KwaZulu-Natal, South Africa. Health Policy Plann. 2011;26(1):53–62. 10.1093/heapol/czq019 20511348

[59] Balcazar H, Alvarado M, Ortiz G. Salud Para Su Corazon (health for your heart) community health worker model: Community and clinical approaches for addressing cardiovascular disease risk reduction in Hispanics/Latinos. J Ambul Care Manage. 2011;34(4):362–372. 10.1097/JAC.0b013e31822cbd0b 21914992 PMC3357063

[60] South African Government. Tobacco Products Control Amendment Act, 2008. In: The Presidency, editor. Cape Town: South African Government, 2009; p. 1–14.

[61] English LM, Hsia J, Malarcher A. Tobacco advertising, promotion, and sponsorship (TAPS) exposure, anti-TAPS policies, and students’ smoking behavior in Botswana and South Africa. Prev Med. 2016;91:S28–S34. 10.1016/j.ypmed.2016.01.014 26824891

[62] Abdel-All M, Putica B, Praveen D, Abimbola S, Joshi R. Effectiveness of community health worker training programmes for cardiovascular disease management in low-income and middle-income countries: A systematic review. BMJ Open. 2017;7(11):e015529. 10.1136/bmjopen-2016-015529 PMC5695434 29101131

[63] Abrahams-Gessel S, Denman CA, Montano CM, et al. Training and supervision of community health workers conducting population-based, noninvasive screening for CVD in LMIC: Implications for scaling up. Glob heart. 2015;10(1):39–44. 10.1016/j.gheart.2014.12.009 25754565 PMC4356016

[64] Coco L, Piper R, Marrone N. Feasibility of community health workers as teleaudiology patient-site facilitators: A multilevel training study. Int J Audiol. 2021;60(9):663–676. 10.1080/14992027.2020.1864487 33403874 PMC8628855

[65] Laurenzi C. Exploring the implementation of a community health worker programme for maternal and child health in the rural Eastern Cape, South Africa. Stellenbosch: Stellenbosch University; 2020.

